# Antibacterial Activity of Nanocomposites of Copper and Cellulose

**DOI:** 10.1155/2013/280512

**Published:** 2013-12-24

**Authors:** Ricardo J. B. Pinto, Sara Daina, Patrizia Sadocco, Carlos Pascoal Neto, Tito Trindade

**Affiliations:** ^1^Department of Chemistry; CICECO, University of Aveiro, 3810-193 Aveiro, Portugal; ^2^Innovhub-SSI Divisione Carta, Via Giuseppe Colombo, 83-20133 Milan, Italy

## Abstract

The design of cheap and safe antibacterial materials for widespread use has been a challenge in materials science. The use of copper nanostructures combined with abundant biopolymers such as cellulose offers a potential approach to achieve such materials though this has been less investigated as compared to other composites. Here, nanocomposites comprising copper nanofillers in cellulose matrices have been prepared by *in situ* and *ex situ* methods. Two cellulose matrices (vegetable and bacterial) were investigated together with morphological distinct copper particulates (nanoparticles and nanowires). A study on the antibacterial activity of these nanocomposites was carried out for *Staphylococcus aureus* and *Klebsiella pneumoniae*, as pathogen microorganisms. The results showed that the chemical nature and morphology of the nanofillers have great effect on the antibacterial activity, with an increase in the antibacterial activity with increasing copper content in the composites. The cellulosic matrices also show an effect on the antibacterial efficiency of the nanocomposites, with vegetal cellulose fibers acting as the most effective substrate. Regarding the results obtained, we anticipate the development of new approaches to prepare cellulose/copper based nanocomposites thereby producing a wide range of interesting antibacterial materials with potential use in diverse applications such as packaging or paper coatings.

## 1. Introduction

The overuse of conventional antibiotics has led to new strains of bacteria with increasing levels of resistance posing potential problems for the public health. There have been efforts from diverse scientific fields in order to achieve solutions that might contribute to attenuate this problem. In this context, research on new bactericidal materials has become a current and important goal in materials science [1]. The development of polymer based nanocomposites with antimicrobial activity offers interesting possibilities because the polymer matrix can be varied in order to fulfill not only specific technological requirements but also nanostructures with size- and shape-dependent properties that can be exploited [2].

Various inorganic nanostructures have been used in a wide range of matrices that result into materials with antibacterial properties [3, 4]. Among the polymer nanocomposites investigated so far, those incorporating Ag and Cu nanoparticles have been regarded as particularly useful for applications in various fields, including biomedical equipment and devices, water treatment, and food processing [3, 5]. Silver has been widely investigated regarding its antimicrobial activity due to its superior effectiveness and strong cytotoxicity towards a broad range of microorganisms, such as bacteria and fungi [1]. The efficacy of Ag NPs as antimicrobial agent is well established and silver based materials have been used in a variety of commercial products. Although there is strong evidence that the antimicrobial activity of silver is associated with cationic release, the mechanism is not totally understood posing some concerns about the potential cytotoxicity and genotoxicity in human cells [6, 7]. In this context, it seems of interest to search for alternatives that could replace, if not totally, at least to some extent silver nanoparticles used as fillers in some composite materials. Copper is of natural occurrence in plant and animal tissues where it participates in a number of important roles. To certain limits, the human body has mechanisms available for protection against copper toxicity at the cellular, tissue, and organ levels [8, 9]. It has been reported that Cu NPs have bactericidal effects comparable to Ag nanoparticles in single strains of *E. coli* and *B. subtilis* [10].

The great interest for Cu based composites can be easily perceived by the number of polymer matrices investigated in their preparation, both of synthetic and natural origin [11]. Among the biopolymers used, the polysaccharides, cellulose [8, 12], starch [13], and chitosan [14], have received special attention. These are renewable polymers with potential biocompatibility and biodegradability that can be used in a variety of formulations depending on the envisaged functionality [15].

Following our own interest in developing silver based antimicrobial materials [16, 17], we report here our first study on the antibacterial activity of bionanocomposites made of copper and bacterial (BC) and vegetable cellulose (VC). This research follows our recent findings that the chemistry of Cu nanostructures, in ambient conditions and when incorporated in cellulose matrices, depends on the morphological features of the copper particles as well as on the type of cellulose employed [12]. Although bacterial and vegetable cellulose are identical from a chemical point of view, their distinct microstructures seem to influence the chemical stability of incorporated copper nanostructures, thereby with potential effects on the antibacterial properties of the corresponding composites. Hence, this research will have focus on the antibacterial activity of nanocomposites in which the cellulose matrices are different and at the same time matching Cu nanofillers with distinct morphologies (spherical NPs and NWs).

## 2. Materials and Methods

### 2.1. Materials

All chemicals were used as received: copper (II) sulphate pentahydrate (p.a., Panreac), copper (II) nitrate trihydrate (p.a., RPE), trisodium citrate (99%, BDH), sodium borohydride (NaBH_4_) (95%, Riedel-de Haën), sodium hydroxide (98,5%, Acrôs Organics), ethylenediamine (99%, Aldrich), and hydrazine hydrate (50–60%, Sigma-Aldrich). Wood cellulose fibers (*Eucalyptus globulus*, ECF bleached kraft pulp, average length 0.9 mm, average width 20 *μ*m) composed essentially of cellulose (~85%) and glucuronoxylan (~15%) were supplied by Portucel (Portugal). Wood cellulose was disintegrated and washed with distilled water before use. Pure bacterial cellulose was produced by *Acetobacter xylinum*, in the form of a wet 3D network of ribbon-like nanofibril structures (50–100 nm width).

### 2.2. Preparation of the Cellulose/Copper NPs Nanocomposites

The preparation of the nanocomposites containing Cu NPs was performed by an adaptation of the procedure described by Loo et al. [18] for Cu hydrosols but by reducing the copper (II) salt in the presence of vegetable or bacterial cellulose fibers. Thus, the fibers were homogeneously mixed with 5 mL of CuSO_4_·5H_2_O (1 × 10^−2 ^M) and 60 mL of a sodium citrate solution (5 × 10^−3 ^M), over 1 hour. This suspension was purged under a N_2_ stream and then 30 mL of NaBH_4_ (2 × 10^−2^ M) and 30 mL of NaOH (2 × 10^−2^ M) were added drop wise, under vigorous stirring. The mixture was then stirred and maintained under N_2_ atmosphere for 1 hour. The nanocomposites acquired a red color after complete copper (II) reduction, after which the product was collected by filtering and thoroughly washed with distilled water. The VC nanocomposites were dried overnight in a desiccator with silica gel and the BC nanocomposites were lyophilized.

### 2.3. Preparation of the Cellulose/Copper NWs Nanocomposites

The copper NWs were prepared by reduction of copper (II) nitrate with hydrazine in alkaline medium [19]. An aqueous solution (1 mL) of Cu(NO_3_)_2_·3H_2_O (0.1 M) was mixed with 20 mL of NaOH (15 M) aqueous solution. This solution was kept under N_2_ and then 150 *μ*L of ethylenediamine and 25 *μ*L of hydrazine were added, by this order, to the reacting mixture. The temperature of this mixture was set to 60°C, with vigorous stirring, for a period of 1 hour.

The cellulose nanocomposites containing the nanowires were prepared by mixing Cu NWs and cellulose (VC or BC) fibers in 20 mL of water. This mixture was kept under constant stirring, at room temperature, over 3 h. The resulting nanocomposites were collected by filtering and thoroughly washed with distilled water. The VC nanocomposites were dried and the BC nanocomposites were lyophilized.

### 2.4. Stock Cultures and Culture Media

All microbial strains cited in the paper were provided by DSMZ, Deutsche Sammlung von Mikroorganismen und Zellkulturen GmbH (German Collection of Microorganisms and Cell Cultures). *K. pneumoniae* ATCC 4352 (DSM 789) and *S. aureus *ATCC 6538 (DSM799) were maintained frozen (−80°C) and transferred monthly on TSA (Tryptone Soya Agar) made of 15 g/L tryptone, 5 g/L soya peptone, 5 g/L NaCl, and 15 g/L neutralized bacteriological agar.

### 2.5. Quantitative Assessment of Antibacterial Activity of Cellulose/Cu Nanocomposites

All bacterial preinoculum cultures were grown overnight at 37°C in 20 mL of Nutrient Broth (made of 1 g/L beef extract; 5 g/L neutralized peptone; 2 g/L yeast extract; 5 g/L NaCl) subjected to horizontal shaking at 100 rpm. The nanocomposite samples were placed in contact with a microbial liquid suspension, subjected to vigorous shaking in order to assure the best contact between bacteria and sample. At 0 h and 24 h contact times, the bacterial concentration (CFU/mL) of the microbial suspension was determined by plating serial dilution on Plate Count Agar to obtain the overall number of bacteria (CFU—Colony Forming Units). For the antibacterial tests the specific conditions were as follows:

(i) microbial liquid suspension: 5 mL of 5% Nutrient Broth in phosphate buffer (0.3 mM, pH 7.2) inoculated with 10^−4^–10^−5^ CFU/mL bacteria;
(ii) total flask volume: 25 mL;
(iii) sample incubation: 24 hours at 23 ± 1°C under vigorous shaking;
(iv) quantity of tested material: 100 mg for vegetable cellulose based samples and 50 mg for bacterial cellulose based samples. The samples were cut in small pieces and tested in duplicate;
(v) control samples: BC and CV fibers without the addition of Cu were tested as blank reference, while as internal reference of the method the bacteria growth was tested on flasks only containing inoculated Broth media. All the samples were subjected to sterilization by autoclave.

The bacteria log reduction of the samples was calculated as follows: log reduction = log (CFU T_24_ control sample) − log (CFU T_24_ nanocomposite). As mentioned in the standard dynamic shake flask method, at least a 1 log reduction of bacteria load is required to claim antibacterial property.

### 2.6. Instrumentation

Scanning electron microscopy (SEM) images were obtained using a Hitachi SU-70 instrument fitted with an energy dispersive spectroscopy (EDX) accessory (EDX Detector: Brüker AXS, Software: Quantax). Samples were deposited on a glass plate and coated with carbon.

The diameter of the Cu nanoparticles was determined by analysis of SEM micrographs of the BC/Cu NPs nanocomposites. In this case, at least 50 NPs were analyzed using ImageJ program and the average value and its standard deviation were calculated, respectively.

The optical spectra were recorded using a Jasco V-560 UV-Vis spectrophotometer; for solid samples the spectra were recorded in the diffuse reflectance mode using MgO as the reference. Inductively Coupled Plasma Optical Emission Spectrometry (ICP-OES), using a Jobin Yvon 70 Plus equipment, was used to determine the copper content. Typically, the samples are digested in a microwave at 160°C with concentrated nitric acid before analysis.

## 3. Results and Discussion

**Figure 1:**
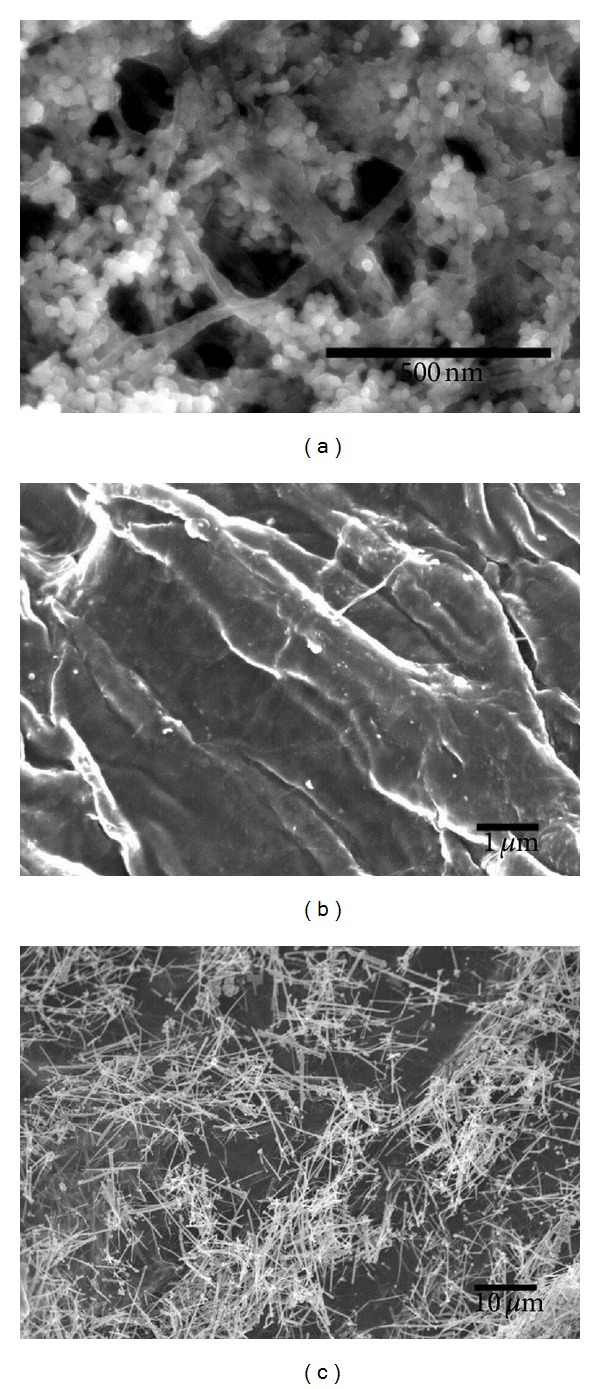
SEM images of (a) BC/Cu NPs, (b) VC/Cu NPs, and (c) BC/Cu NWs nanocomposites.

A number of Cu/cellulose nanocomposites have been prepared using both VC and BC fibers as the matrices. Typically the nanocomposites with Cu NPs were obtained by the *in situ* reduction of Cu(II) and Cu NWs composites analogues were obtained by blending the previously prepared nanowires with the cellulose matrices. Vegetable cellulose was not effective in the formation of *ex situ* nanocomposites. The BC matrix formed an intimate mixture with the Cu NWs because the nanofibrils had the ability to be detached from the matrix by rolling up the Cu NWs. SEM images (Figure 1) show the morphological characteristics of the distinct cellulose/Cu nanocomposites investigated in this research. The BC/Cu NPs nanocomposites (Figure 1(a)) present a homogeneous distribution of well-defined spherical Cu NPs (ca. 36 nm diameter) clustered at the nanofibers surfaces. For the VC nanocomposites (Figure 1(b)), the images show a film over the cellulose fibers probably consequence of the oxidation of the NPs on the surface of this biopolymer. The BC/Cu NWs nanocomposites (Figure 1(a)) show Cu NWs with diameters in the 90–220 nm range and micrometric lengths blended with BC nanofibrils.

**Figure 2:**
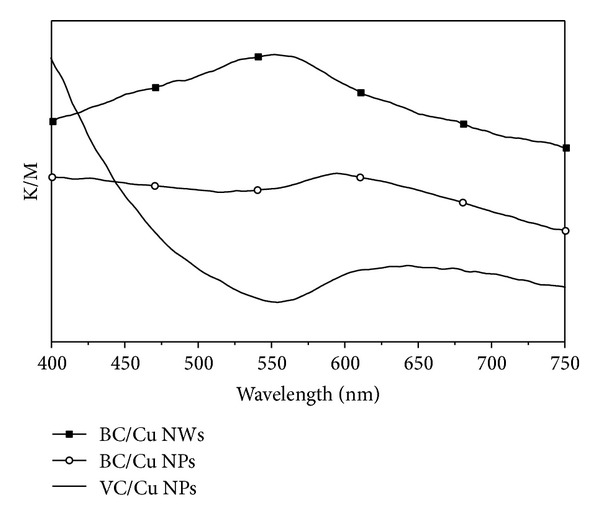
Optical spectra of as-prepared cellulose/Cu nanocomposites (Kubelka-Munk data).

Figure 2 shows the optical spectra of the cellulose/copper nanocomposites. Figure 2 shows that the nanocomposite prepared with BC fibers maintained the characteristic surface plasmon resonance (SPR) band in the visible region (568 nm) assigned to Cu nanoparticles [20], though a slight red-shift was observed as compared to the Cu aqueous colloid. As discussed in our previous work [12], this shift can be explained by changes in the dielectric function of the surrounding medium (from water to cellulose). The CV/Cu nanocomposite does not exhibit the SPR band which has been explained on the basis of the Cu nanostructures oxidation when exposed to air, thus confirming the results observed in Figure 1(b). These differences are in line with the color observed for all cellulose/copper nanocomposites; thus the BC nanocomposites maintained the characteristic reddish hue of metallic copper while the VC composites acquired a greenish color shortly after their synthesis, due to oxidized copper species [12].

The results above have been explained as consequence of a less chemical stability of Cu NPs grown on VC fibers against oxidation as compared to those present in BC fibers [12]. In fact, although having an identical molecular structure, the VC fibers form a more open structure in which permeation to oxygen is higher than in the case of BC, thus facilitating the oxidation of Cu NPs attached to the fibers surfaces [12].

In order to evaluate the antibacterial activity of the cellulose/Cu nanocomposites, selected samples have been tested towards bacteria strains of *Staphylococcus aureus* and *Klebsiella pneumoniae*.  Table 1 lists the selected nanocomposites together with the respective Cu content as determined by elemental analysis using ICP.

**Figure 3:**
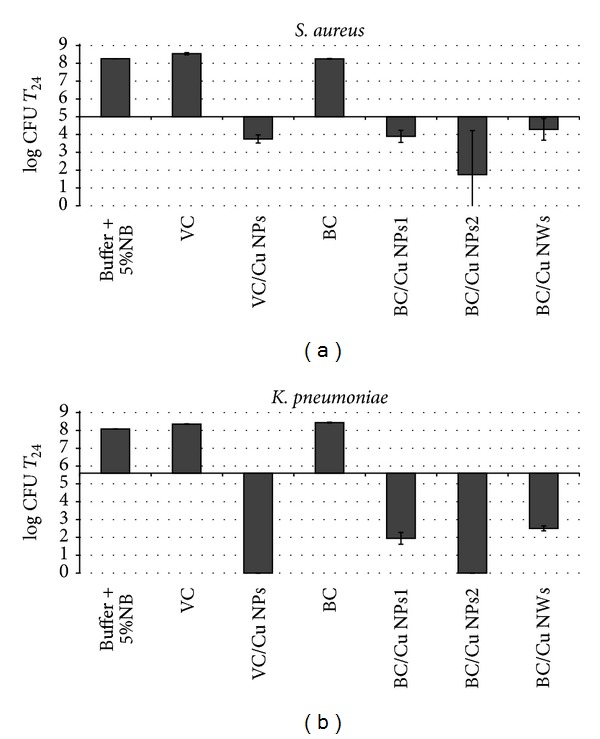
Antibacterial activity of cellulose/Cu nanocomposites with variable Cu content against (a) *S. aureus* and (b) *K. pneumoniae*. The log CFU values were determined in the testing Broth after 24 h contact time and were compared to those of pure cellulose matrices as well as to the inoculated Broth alone. Horizontal dark line refers to the initial inoculum (log CFU at time 0).

Figure 3 shows the results obtained for the antibacterial activity of the distinct nanocomposites and for blank cellulose matrices used as control. For both bacteria strains the control samples did not exhibit antibacterial activity (value of log CFU at T_24_ for control samples and the buffer solution was almost identical).

Conversely, the antimicrobial tests revealed that the Cu nanocomposites have antibacterial action for both bacteria, though with a more pronounced effect in respect to *K. pneumoniae*. Although there is some debate in the literature about the relative effect of nanoparticles on the type of bacteria, this study is in line with reports that suggest that Gram-negative bacteria are more affected by copper based materials [3]. In Gram-negative bacteria, peptidoglycan layer is thinner (between 2 and 3 nm) than Gram-positives (around 30 nm) and externally to this layer there is an outer asymmetric membrane composed of proteins, phospholipids, and lipopolysaccharides [1]. Some authors have explained the higher antibacterial effect in Gram-negatives bacteria as consequence of interactions occurring between the bacteria outer membrane and solid surfaces, either for nanoparticles of copper [3, 21] or of silver [3, 22].

Such particle-microorganism interaction promotes the formation of irregular pores in the outer membrane of Gram-negative bacteria, due to direct interaction of the nanoparticles or metallic ions released, changing its permeability and causing the release of cell components. These structural changes result in the membrane degradation and eventually in the death of the bacteria [22, 23].

Several studies have reported that the extent of inhibition of bacterial growth in this type of materials depends on the inorganic content in the medium, both for the case of metal NPs and for metal oxides used as fillers [16, 24]. For the nanocomposites investigated here two samples spherical Cu NPs (BC/Cu NPs1 and BC/Cu NPs2) have been evaluated by varying the Cu content. The increasing of Cu content from 0.93 to 4.95% (w/w) resulted in a significant bactericidal effect (about 2 log bacterial growth over 24 h incubation time) against the *S. aureus*. A similar trend was also observed for the *K. pneumoniae* strain, verifying a direct dependence of the antibacterial action with the of Cu content in the composite. Note that complete killing effect was observed for the BC/Cu NPs2 nanocomposite. Similarly to silver materials, the antibacterial activity of Cu nanostructures has been associated with the release of ionic species and the formation of reactive oxygen species [1, 5]. The increase of the copper amount in the nanocomposites results in a higher release of cations, increasing in this way the antibacterial activity of the corresponding cellulose based nanocomposites.

It is interesting to note that despite the influence of copper content, the sample with the higher copper content (BC/Cu NWs sample) did not present the higher antibacterial effect. For both bacteria studied, BC/Cu NWs nanocomposite present an antibacterial activity significantly lower (more than 2 log bacterial growth) in relation to the nanocomposite with copper NPs with similar copper amount (BC/Cu NPs2). Even BC/Cu NPs1 nanocomposite presents slightly higher antibacterial efficiency, this composite presenting 5 times less copper amount. Assuming that the antibacterial effect is mainly due to cationic release, this lower efficiency for the Cu NWs containing composites, as compared to those incorporating Cu NPs, is probably due to the less surface reactivity of the nanowires, thus leading to lower amounts of soluble and oxidized copper species. In fact, this explanation is in agreement with the observations presented above for the easier oxidation of the Cu NPs as compared to that of the Cu NWs. In fact, the former materials have already oxidized copper phases and the surface chemistry is thus markedly distinct from the Cu NWs.

There is lack of knowledge about the effect of the morphology of copper nanostructures on their antibacterial properties, both in the case of individualized nanoparticles and when used in composite materials. However, studies performed on silver nanostructures with distinct morphologies have demonstrated that Ag NPs undergo a shape-dependent interaction with the bacteria [25]. Through the study of bacteria surface by transmission electron microscopy, the authors found that spherical NPs exhibit enhanced antibacterial activity than, for example, Ag nanorods. This effect was explained by the higher reactivity of these nanostructures because of higher atomic density. In this case, to obtain similar antibacterial activity of 12.5 mg of spherical NPs were necessary 50–100 mg of nanorods. The higher reactivity of the NPs probably leads to a faster release of metallic ions leading to an enhancement of the antibacterial activity for this type of nanostructure.

Finally the effect of the cellulose matrix should also be noted. For this analysis 100 mg of the VC/Cu NPs nanocomposite is used and 50 mg of BC/Cu NPs1 so the total amount of inorganic filler is almost the same. For *S. aureus* the VC/Cu NPs nanocomposite presents a similar activity to the BC/Cu NPs1 composite. However, comparing the same nanocomposites for the antibacterial action against *K. pneumoniae*, the nanocomposite prepared with VC fibers shows a superior effect. This is an unexpected observation because the Cu NPs on the VC fibres are not individualized but forming a film, as already reported [12], due to the fast oxidation under normal ambient conditions. Probably this is explained by the preferential deposition of copper on the VC fibers' surfaces which contrasts to the BC matrix in which the NPs also rely on the BC structure due to its three-dimensional internal organization, thus acting as a protective cage for the Cu NPs. In this case, the release of Cu ions is limited as compared to the more open structure of the CV nanocomposite. Similar observations have been reported for materials based on cellulose and Ag NPs, in which for VC/Ag nanocomposites the release of Ag^+^ was superior compared to the BC analogues [16].

## 4. Conclusions

A series of cellulose/copper nanocomposites have been prepared by varying the type of cellulose used as the matrix (vegetable or bacterial) and also the morphology of copper nanostructures (nanoparticles or nanowires) used as fillers. These composites were investigated for the first time for their antibacterial activity. Antibacterial activity has been observed for the nanocomposite samples against both Gram-positive (*S. aureus*) and Gram-negative (*K. pneumoniae*) bacteria. Enhancement of the antibacterial activity with increasing copper content was observed. Among the morphological distinct copper nanostructures used, the nanowires have shown less antibacterial effect that was ascribed to the less reactive surface towards oxidation. Another parameter that influences the antibacterial efficiency of the nanocomposite was the structure of the cellulose fibers. The results suggest that the use of copper together with vegetable cellulose fibers results in better antibacterial materials against both species of tested bacteria. These results confirm the potential of bionanocomposites containing copper nanostructures as new antimicrobial materials. Furthermore this study has shown that formulating composites in which both the matrix and the morphological characteristics of the Cu fillers are varied can improve the antibacterial action of such materials. These parameters seem to influence the antimicrobial mechanism present that although consistent with a cationic release process still needs further evidence.

## Figures and Tables

**Table 1:** Selected cellulose/copper nanocomposites used in the antimicrobial essays.

Cellulose substrate	Code	Cu (% w/w)
Vegetable cellulose (VC)	VC	—
	VC/NPs	0.55

Bacterial cellulose (BC)	BC	—
	BC/NPs1	0.93
	BC/NPs2	4.95
	BC/NWs	5.17
